# Supplementary material for: High-throughput mechanomic screening reveals novel regulators of single-cell mechanics
Source: Biophys J. 2025 Oct 10;125(12):3060–73. doi: 10.1016/j.bpj.2025.10.008 (PMC13507181; doi:10.1016/j.bpj.2025.10.008)
Supplement: Supplementary file 1 [file mmc1.pdf]

Biophysical Journal, Volume *125*

Supplemental information

High-throughput mechanomic screening reveals novel regulators of single-cell mechanics

Laura von Selzam, Katarzyna Plak, Christine Schweitzer, Cornelia Liebers, Paul Müller, Marta Urbanska, Martin Kräter, Buzz Baum, Jona Kayser, and Jochen Guck

SUPPLEMENTAL RESULTS

Additional full population knockdown effects caused by mitotic fraction change

Besides the 58 mechanical hits shared with the cell cycle-resolved analysis, the full population analysis returned 12 additional full population hits which did not cause any significant mechanical effect in either interphase or mitosis (Fig. S1A). All these genes caused relatively small stiffening effects in the full population analysis (compare Table S3). Since all these full population exclusive hits were associated with increased stiffness, we hypothesized that their full population effects may reflect knockdown-induced shifts in cell cycle distribution rather than genuine changes in single-cell stiffness. Since mitotic cells are inherently stiffer than interphase cells, an increase in the number of mitotic cells upon gene knockdown could artificially increase the mean Young's modulus in the full knockdown population. A illustrative example for this process is provided by the knockdown of *CG32812* (Fig. S1B), where an increased mitotic fraction in the knockdown sample shifts the mean stiffness of the full population upwards, while the Young's modulus means in the interphase and mitotic subpopulation remain almost unchanged.

To test whether increased mitotic fractions generally correlated with apparent stiffening effects, we compared changes in mitotic fraction across all identified mechanical hits (Fig. S1C). Genes identified as mechanical hits in both full population and cell cycle-resolved analyses typically showed a 4% increase in mitotic cells compared to controls. In contrast, hits exclusively identified through cell cycle-resolved analysis exhibited a significantly smaller increase in mitotic fraction of around 2%. Genes which only influenced mechanics in the full population showed the largest mitotic fraction increases of around 7%, with only one gene (*Abl*) exhibiting a decreased mitotic fraction upon knockdown. These findings support our hypothesis that knockdown-induced increases in mitotic cell numbers, rather than changes in individual cell stiffness, contributed to many of the apparent mechanical phenotypes in the full population analysis.

To directly test this hypothesis, we performed a mitotic downsampling reanalysis of our data (Fig. S2). For each full population hit exhibiting increased mitotic fractions, we reduced the number of mitotic cells in the knockdown samples by randomly removing mitotic cells until the mitotic fraction matched that of the corresponding control. We then repeated the full population analysis to determine whether the previously observed mechanical phenotypes persisted even without the influence of increased mitotic fractions (Fig. S1D). After correcting for mitotic fraction shifts via mitotic downsampling, 10 out of the 12 genes exclusively identified in the full population analysis no longer showed significant stiffening effects, with only *Abl* and *PpD5* remaining significant.

SUPPLEMENTAL DISCUSSION

The knockdown effect of *PpD5* was both weaker and less significant after downsampling. *Abl* could not be downsampled as its knockdown sample already had a lower number of mitotic cells compared to the control. However, since the p-value of *Abl* was close to the significance threshold in both the full-population and cell cycle-resolved analyses, it is likely that its small mechanical effect reached statistical significance only due to the larger sample size in the full-population analysis, while remaining undetectable within the smaller interphase and mitotic subpopulations. The same explanation likely applies to the weakened significance of *PpD5* following downsampling. When applied to the mechanical hits from the cell cycle-resolved analysis (see Fig. 1C), all genes except *HINT1*, which showed weak stiffening upon knockdown, retained their significance (details in Table S2). These results demonstrate that most stiffening effects observed exclusively in the full population analysis can be attributed to shifts in cell cycle distribution instead of genuine changes in single-cell mechanics.

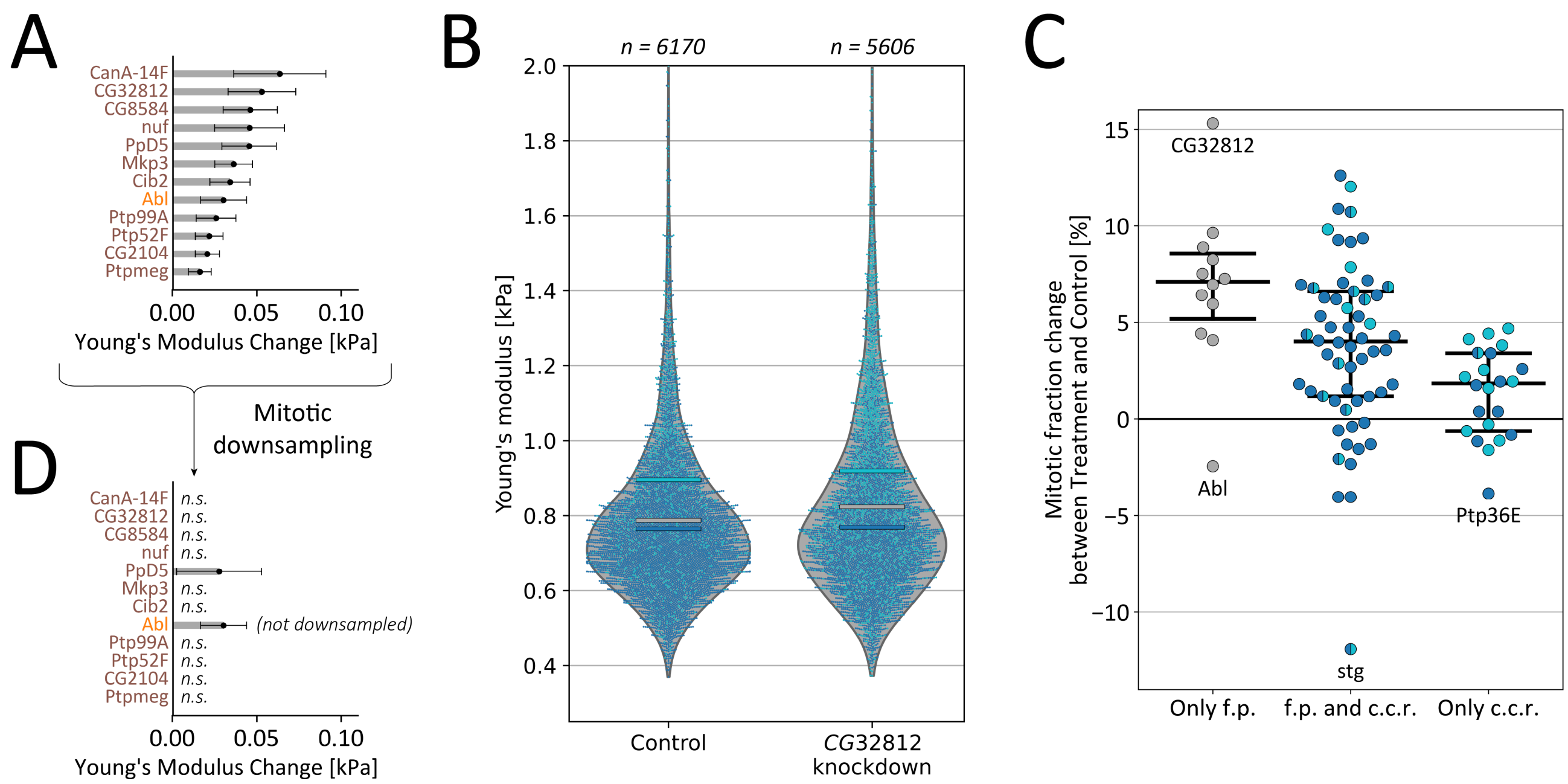

Figure S1: Additional hits in the full population analysis are caused by an increased mitotic fraction. **A** Knockdown effects of genes which exclusively change the mean Young's modulus of the full cell population. Phosphatase genes are labeled in brown, kinase genes in orange. **B** Representative Young's modulus distributions for full population exclusive hits, measured in *CG32812* knockdown cells and their matched daily control sample. Each dot represent a single cell measurement, with the blue dots representing cells in interphase and cyan dots representing cells in mitosis. Horizontal lines represent mean Young's modulus for the full cell population (gray), as well as for the interphase (blue) and mitotic (cyan) subpopulations. For the control sample, 15 measurements are above the plot window going up to 5.4 kPa. For the knockdown sample, 31 measurements are above the plot window going up to 5.6 kPa. **C** Difference in mitotic fraction $n_{\text{mitotic}}/n_{\text{total}}$ between gene knockdown and control samples , categorized by the analysis method that identified them as the hits, f.p. for full population (see Fig. S1A), c.c.r. for cell cycle-resolved (see Fig. 2A for f.p. and c.c.r., Fig. 2B for only c.c.r). Outliers are labeled with their gene symbol. **D** Knockdown effects of full population exclusive hits after mitotic downsampling. Genes with a mitotic fraction change < 0 were not downsampled. Significance testing for A and D was performed through linear mixed model analysis.

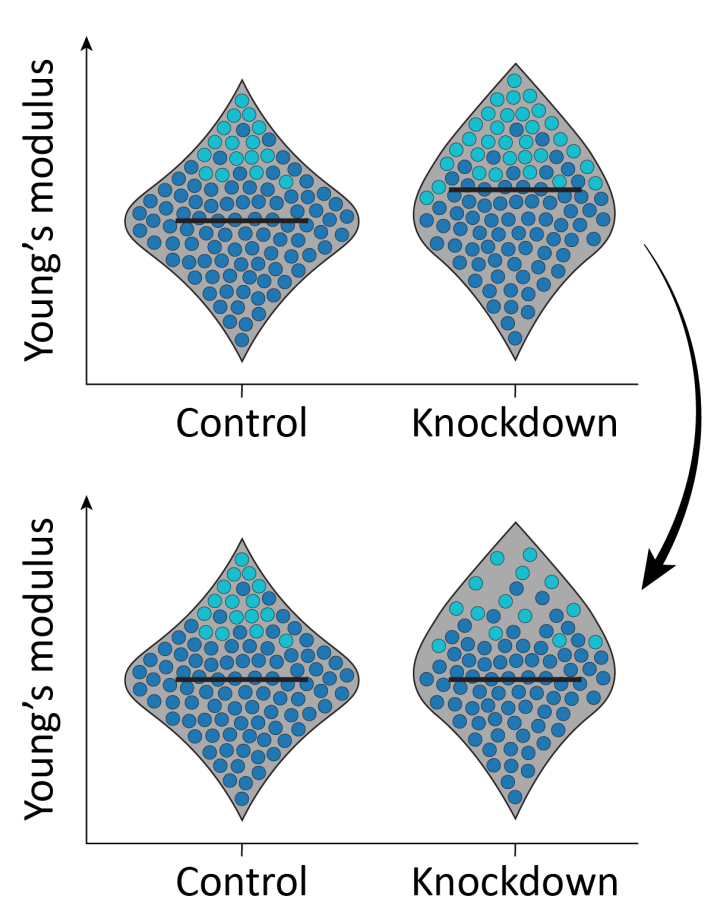

Figure S2: Schematic illustration of the mitotic downsampling workflow.
